# Nanoscale Mechanical Properties and Indentation Recovery of PI@GO Composites Measured Using AFM

**DOI:** 10.3390/polym10091020

**Published:** 2018-09-13

**Authors:** Ji Zhou, Qiang Cai, Fu Xu

**Affiliations:** 1 College of Civil Engineering, Hunan University of Science and Engineering, Yongzhou 425006, China; hnkjxy_zhouji@163.com; 2 College of Civil Engineering and Mechanics, Xiangtan University, Xiangtan 411105, China; caiqiangvip@foxmail.com

**Keywords:** polyimide, graphene oxide, composite, mechanical properties, indentation recovery, AFM

## Abstract

Polyimide@graphene oxide (PI@GO) composites were prepared by way of a simple solution blending method. The nanoscale hardness and Young’s modulus of the composites were measured using nanoindentation based on atomic force microscopy (AFM). A nanoscale hardness of ~0.65 GPa and an elastic modulus of ~6.5 GPa were reached with a load of ~55 μN. The indentation recovery on the surface of PI@GO was evaluated. The results show that relatively low GO content can remarkably improve the nanoscale mechanical properties of PI.

## 1. Introduction

Polyimide (PI) is a well-known high-performance polymer that has excellent thermal, mechanical, and electrical properties, as well as outstanding chemical resistance [1,2]. PI products are widely used in defense and aerospace applications, as well as in the electronics industry, for a variety of interconnect and packaging applications. Nowadays, electronic products require the PI to possess a high glass transition temperature and better thermal mechanical strength. To address this concern, PI-based composites with various fillers including carbon nanotubes [3], SiC, graphene [4], graphene oxide [5], SiO_2_ [6], and aramid fibers [7] have been explored. The composites are highly affected by the reinforcements, in which the dimension, dispersion state, and the interaction of the reinforcements play significant roles. Actually, the aggregation of nanosize fillers easily results in performance deterioration of the composites. Thus, the preparation of uniformly dispersed PI-based composites is critical to the applications. Among the nanosize fillers, graphene oxide (GO) nanosheets have attracted much attention due to their high dispersibility, easy preparation, and low cost.

Recently, PI@GO composites have been prepared by various methods such as in situ polymerization [8], chemical cross-linking [9], and thermal imidization [10]. Dynamic mechanical analysis indicates that the storage modulus and the glass transition temperature of PI are improved by addition of GO. Besides this, the thermal stability of PI is enhanced with increasing GO content [10]. However, most of the previous studies focused on the macroscopic properties of PI@GO composites [11,12] while, actually, the nanoscale properties of PI-based composites are crucial to their applications in aerospace power and propulsion components. Unfortunately, only limited methods are available to probe the nanomechanical performance of polymer composites [13,14,15]. Indentation analysis is used to determine the mechanical properties of an indented material by plotting the indentation force versus depth, and has been well established for homogeneous materials on Hysitron or MTS systems. However, the load resolution and size limitations of the indenter restrict its application [16]. Relative to indentation analysis, atomic force microscopy (AFM) is considered more accurate at nanoscale due to the smaller tip radius and indentation depth, which eliminate the effects of adhesion and plastic deformation on the measurement. Herein, PI@GO composites were prepared by way of a simple solution blending method. The nanoscale mechanical properties and indentation recovery of the composites were measured using nanoindentation based on AFM.

## 2. Materials and Methods

Soluble PI powders (Matrimid@ 5218, *M*_w_ = 80,000) were purchased from Zhuzhou Time New-Materials Tech. Co, Ltd. (Zhuzhou, Hunan, China). Dimethylacetamide (DMAc) was purchased from Tianjin Hengxing Chemical Reagent Co, Ltd. (Tianjin, China). GO used in our research was purchased from Suzhou Tanfeng Tech. Co, Ltd. (Suzhou, Jiangsu, China). All materials are commercially available and were used without further purification. A certain amount of PI was dissolved in the DMAc, then an amount of GO (0.1 wt % based on the weight of PI) was ultrasonically dispersed into the PI solution. Then, the as-prepared solution was transferred into a metal mold, followed by drying in vacuum at 100 °C for 48 h under a pressure of 4 mbar. After the solvent was evaporated, PI@GO composites were obtained. For comparison, a pure PI sample was prepared analogously to the PI@GO. Before being submitted to AFM testing, the PI and PI@GO samples were cut into 1 × 1 cm pieces.

The mechanical properties of the PI@GO composites at nanoscale were characterized using AFM (Bruker, Santa Barbara, CA, USA). Nanoindentation was performed with AFM at three different areas by using a hand-crafted natural diamond tip with a spring constant of ~225 N/m (Bruker, Santa Barbara, CA, USA). The height of the tripyramidal tip is 50 μm, and the front, back, and side angles of the tripyramid are 55°, 35°, and 51°, respectively. The tip radius is 50 nm. The morphology of the samples was examined by using a common silicon nitride tip (Bruker, Santa Barbara, CA, USA).

**Figure 1:**
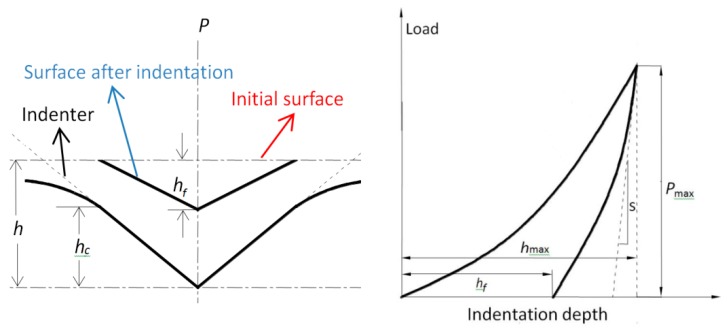
Schematic diagram of a typical indentation.

In a typical AFM indentation test, a controlled diamond tip is driven into the specimen surface, and the displacement of the indenter tip is continuously monitored by high-resolution sensors. Various mechanical properties (most typically the elastic modulus and hardness) of the indented material can be measured by analyzing the indentation data. The Oliver–Pharr model was employed to evaluate the hardness and Young’s modulus in this paper. A schematic diagram of a typical indentation is shown in Figure 1. The indentation hardness proposed by Olive and Pharr is defined as [17]

(1) $$H=\frac{P_{\max}}{A}$$

where $P_{\max}$ is the maximum load and A is the projected area of the indentation. The projected contact area is linked to the contact depth $h_{c}$ by the tip geometry. In case of a nonideal probe, the deviations of the contact area can be expressed in the form of a fitting function as shown below [18]:

(2) $$\begin{array}{c} A=24.5h_{c}^{2}+793h_{c}+4238h_{c}^{1/2}+332h_{c}^{1/4}+0.059h_{c}^{1/8}\\ +0.069h_{c}^{1/16}+8.68h_{c}^{1/32}+35.4h_{c}^{1/64}+36.9h_{c}^{1/128} \end{array}$$

(3) $$\mathrm{with}\;h_{c}=h-\varepsilon \frac{P_{\max}}{S}$$

where *h* and *s* are the indent depth and contact stiffness, respectively. ε is a constant that depends on the indenter geometry (ε = 0.75 for a Berkovich indenter).

Contact stiffness *S* and Young’s modulus *E* of the material can be expressed as

(3) $$\frac{1}{E_{r}}=\frac{1-\nu^{2}}{E}+\frac{1-\nu_{i}^{2}}{E_{i}}$$

(4) $$S=\frac{dP}{dh}=\frac{2}{\sqrt{\pi }}E_{r}\sqrt{A}\;$$

where $E_{r}$ is the reduced elastic modulus, which accounts for the fact that elastic deformation occurs in both the sample and the indenter. E and ν are the elastic modulus and Poisson’s ratio for the sample, respectively. $E_{i}$ and $\nu_{i}$ are the same quantities for the indenter. For diamond, $E_{i}$ = 1141 GPa and $\nu_{i}$ = 0.07 [19,20].

## 3. Results and Discussion

**Figure 2:**
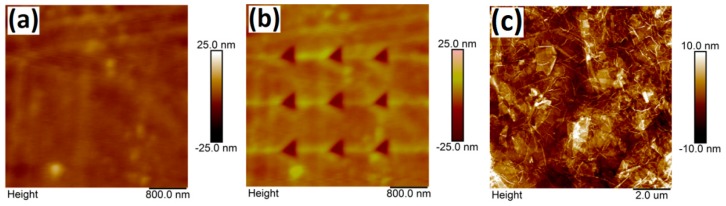
Typical topographical features of the polyimide@graphene oxide (PI@GO) sample before (**a**) and after (**b**) indentation tests. (**c**) AFM image of GO nanosheets.

A diamond cone tip was used as the indenter, and its sensitivity was calibrated using a sapphire standard specimen. Typical topographical features of the PI@GO sample before and after indentation tests are shown in Figure 2. A relatively flat surface (*R*_q_ = 2.24 nm) can be observed before AFM nanoindentation (Figure 2a). After capturing the morphology, the ramp mode was employed to perform the nanoindentation. A 3 × 3 array of indentations was conducted at different indent forces. Each residual indent was imaged by the indenter in tapping mode immediately after the indentation and later by a new and sharp silicon tip in tapping mode. As shown in Figure 2b, all AFM images of the three indents scanned with the indenter show the same triangular shape that is similar to the expected cross section of a three-sided pyramidal tip. The shapes and sizes of the three indentations under the same loading level are highly consistent with each other. No detectable pile-up or significant crack along the edges is observed, indicating that the applied load is not high enough to drive cracks. Due to the homogenous dispersion of GO in the matrix, no nanosheets can be observed in the AFM image. Figure 2c shows the morphology of the GO nanosheets. The lateral size of the GO sheets is in the range of 200 nm ~ 3 μm. The thickness of a single-layer GO nanosheet is about 1.2 nm.

**Figure 3:**
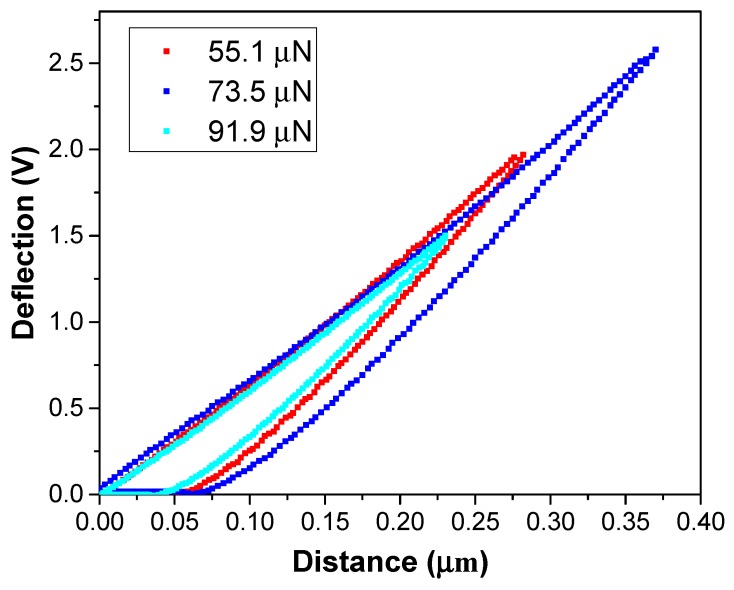
Typical deflection–distance curves of the PI@GO composite.

Figure 3 shows typical deflection–distance curves of the PI@GO composite under a load of up to ~91.9 μN. A deflection–distance curve recorded by AFM can be converted to a force–indentation plot. The force can be calculated by multiplying the cantilever deflection with calibration factors that have been experimentally determined [21]. Here, the voltage setpoints 1.5, 2.0, and 2.5 V correspond to loading forces 55.1, 73.5, and 91.9 μN, respectively. As shown in the loading curve, the deflection increased linearly with increasing indenting force. The unloading and loading curves are not overlapped. Resident depth can be measured from the deflection–distance curve. Based on these measurements, it was assumed that the indenter is a rigid body.

**Figure 4:**
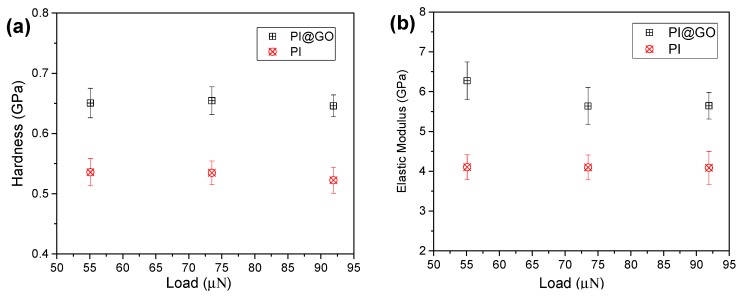
Hardness (**a**) and elastic modulus (**b**) of PI and PI@GO composite under different loads.

The hardness and elastic modulus of PI and PI@GO composite were calculated as shown in Figure 4. The hardness of the PI under different loading forces is in the range of 0.5~0.56 GPa (Figure 4a). The hardness of PI does not show an obvious decrease with the increase in indent force, indicating that no size effect is presented in the hardness. The reason for the lack of noticeable size effect might be that the three load forces are relatively close to each other. It was reported that monolayer graphene oxide has an effective Young’s modulus of ~200 GPa [22]. With the addition of GO nanosheets, the hardness of PI@GO was boosted remarkably. The homogeneous dispersion of GO nanosheets is critical to the enhancement of the mechanical properties of PI. Compared with graphene, the presence of oxygen functional groups makes GO compatible with a polymer matrix. As we expected, the PI@GO composite exhibits a higher elastic modulus compared with pure PI (Figure 4b). The GO nanosheets in the PI matrix effectively resist the penetration of the indenter, resulting in the higher elastic modulus. Robust GO sheets can prevent crack initiation and propagation very well.

**Figure 5:**
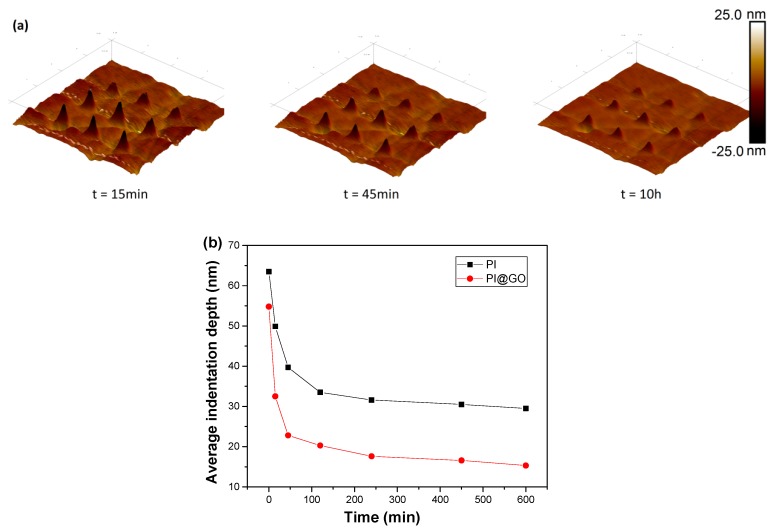
(**a**) AFM images of the indentation recovery of PI@GO. (**b**) Indentation recovery data of PI and PI@GO under the load of 91.9 μN.

The indentation recovery of polymer surfaces has been widely studied in the past decades [23]. The evaluation of indentation topography enables a definitive evaluation of the indentation hardness characterization of polymers and a quantitative determination of the viscoelastic recovery at deformed surfaces. Yanhuai Ding et al. discussed the time-dependent viscoelastic recovery of indentations on a polymethyl methacrylate surface [24]. A simple Kelvin model was employed to predict the indentation recovery. As shown in Figure 5a, the inverse AFM images clearly demonstrate the morphology changes of indentations on the PI@GO surface with increasing recovery time. The residual indentation depth was measured using a sharp AFM tip and is summarized in Figure 5b. A nonlinear dependence between the residual indentation depth and time can be observed. The recovery rate decreased dramatically over time. After several hours, the residual depth remained virtually unchanged, which is well known as the plastic deformation. By comparison, PI@GO composites show a higher recovery rate than does PI, which can be ascribed to the excellent mechanical properties of the additives. The imbedding of GO sheets in the polymer matrix accelerated the indentation recovery by releasing the deformation energy [25]. The results indicate that two-dimensional nanomaterials can boost the indentation recovery due to their unique morphological characteristics.

## 4. Conclusions

In summary, the nanoscale hardness and elastic modulus of PI@GO composites were characterized by way of AFM nanoindentation. The indentation recovery of the PI@GO surface was measured compared with that of raw PI. The results show that the hardness and elastic modulus of the PI are enhanced by the addition of GO. The imbedded GO sheets in the polymer matrix accelerate the indentation recovery by releasing the deformation energy. This work demonstrates that 2D nanomaterials can improve the self-healing performance of polymer composites.
